# Metabolic pathway for the universal fluorescent recognition of tumor cells

**DOI:** 10.18632/oncotarget.18551

**Published:** 2017-06-16

**Authors:** Ana Fernandez-Carrascal, Manuel Garcia-Algar, Moritz Nazarenus, Alicia Torres-Nuñez, Luca Guerrini, Neus Feliu, Wolfgang J. Parak, Eduardo Garcia-Rico, Ramon A. Alvarez-Puebla

**Affiliations:** ^1^ Department of Physical Chemistry and EMaS, Universitat Rovira i Virgili, Tarragona, Spain; ^2^ Karolinska Institutet, Stockholm, Sweden; ^3^ Philipps-Universität Marburg, Fachbereich Physik, Marburg, Germany; ^4^ Department of Medical Oncology, Hospital Universitario HM Torrelodones, Torrelodones, Madrid, Spain; ^5^ ICREA, Barcelona, Spain

**Keywords:** circulating tumor cells, breast cancer, optical sensing, glucose uptake, hyperoxia

## Abstract

Quantification of circulating tumor cells (CTCs) in blood samples from cancer patients is a non-invasive approach to monitoring the status of the disease. Most of the methods proposed in the recent years are phenomenological and rely on the use of antibodies labelled with fluorophores, magnetic particles, or immobilized on surfaces to capture the CTCs. Herein, we designed and optimized a method that employs a glucose analogue labelled with a fluorophore which takes advantage of the different metabolic pathways of cancer cells to discern them from normal ones. Notably, we demonstrate that fluorescence signal in tumor cells can be greatly maximized by applying hyperoxia conditions without damaging the cells. These results are demonstrated by means of confocal fluorescence and flow-cytometry measurements in peripheral blood mononuclear cells (PBMC) extracted after Ficoll of human blood samples and spiked with a known concentration of MCF-7 tumor cells.

## INTRODUCTION

Quantification of circulating tumor cells (CTCs) in blood samples from cancer patients is a non-invasive approach to monitoring the status of the disease. In recent years, many devices and methods have been proposed for the detection and quantification of CTCs [1–4]. Notably, most of these methods rely on the use of antibodies labelled with fluorophores, magnetic particles, or immobilized on surfaces to capture the CTCs. However, the number of known antibodies (or aptamers) targeting specific membrane receptors of cancer cells is still very limited, which includes the HER family (EGFR, HER2 and HER3), GD2, PSAm, and PDGF [5]. Thus, most of the methods, including the only FDA-approved (CellSearch^®^, Janssen Diagnostics), uses antibodies against epithelial receptors such as the epithelial cell adhesion molecule (EpCAM) [6, 7]. It is well known that, in metastatic cancer, tumor cells experience the epithelial-mesenchymal transition (EMT). In the EMT, epithelial cells lose their cell polarity and cell-to-cell adhesion, while gaining migratory and invasive properties of mesenchymal stem cells [8, 9]. In this situation, a significant fraction of the CTCs does not express any residual epithelial receptors and, thus, it remains undetected [7, 8, 10]. Furthermore, the loss of epithelial characteristics (cytokeratins, for example) is often described as EMT. In these situations, CTC detection based on predefined markers excludes most populations and tumor phenotypes as a consequence of the intratumoral heterogeneity. A solution to this drawback relies on the use of the intrinsic metabolic properties of tumor cells that differ from those of the healthy ones. Specifically, the Warburg effect describes the increase of aerobic glycolysis and glucose uptake in cancer cells [11]. This phenomenon is, for instance, exploited in the positron emission tomography (PET) imaging of tumors in patients by utilizing 2-[^18^F]fluoro-2-deoxy-D-glucose (^18^F-FDG) [12]. This radioactively-labelled glucose analogue is internalized by tumor cells in much higher quantities than by normal cells. The PET signal from the tumor cells consequently is higher than in the surrounding tissues and, thus, this can be used to determine the position of the tumor within the body. Similarly, *in vitro* assays have been developed, exploiting the higher internalization rate of nanoparticles by invasive cells as compared to non-invasive ones [13, 14].

In this paper, we designed and optimized a method that employs a glucose analogue labelled with a fluorophore 2-[N-(7-nitrobenz-2-oxa-1,3-diazol-4-yl)amino]-2-deoxy-D-glucose (2-NBDG) [15]. Due to higher glucose uptake, the fluorescence signal of tumor cells is significantly larger than that of healthy ones, which allows their discrimination and quantification by standard flow-cytometry. Notably, the signal difference was maximized under high oxygen level conditions (i.e., hyperoxia). For this study, we compared peripheral blood mononuclear cells (PBMC), extracted after Ficoll of human blood samples, with MCF-7 tumor cells. MCF-7 are human epithelial breast cancer cells which have been widely used for breast cancer research, especially for their expression of the estrogen receptor [16]. Furthermore, the expression of both, the glucose transporter 1 (GLUT1) and the EGFR receptor, is elevated in MCF-7 as compared to normal cells [17, 18].

## RESULTS AND DISCUSSION

**Figure 1:**
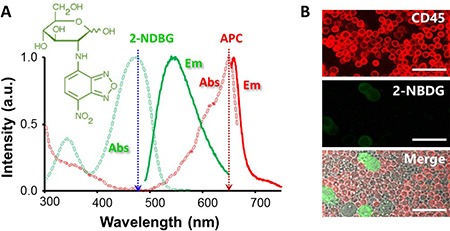
(**A**) Absorption and emission profiles of 2-NBDG and APC. Dotted arrows indicate the excitation lines. Molecular structure of 2-NBDG. (**B**) Confocal fluorescence microscopy images of 2-NBDG uptake for PBMC and MCF-7 incubated with 300 μM 2-NBDG for 30 minutes in samples containing cell ratios of 1:10 MCF-7:PBMC. The bars indicate 40 μm.

The 2-[N-(7-nitrobenz-2-oxa-1,3-diazol-4-yl)amino]-2-deoxy-D-glucose (2-NBDG) is a commercial, non-toxic fluorophore characterized by a quantum yield of 0.55 and a blue absorption at 465 nm, which generates an intense emission at 540 nm upon excitation with a blue laser line (Figure 1A) [19]. After incubation with this fluorophore, normal PBMCs and tumor cells (MCF-7) displayed differences in fluorescence intensity which are too small to univocally distinguish the various types of cells (Figure 1B). Note that to identify PBMCs, samples were also incubated with the anti-leukocyte common antigen (CD45) labelled with allophycocyanin (CD45-APC). APC is a fluorophore characterized by a quantum yield of 0.68 and a red absorption centered at 650 nm, which yields an intense emission at 660 nm upon excitation with a red laser line (Figure 1A) [20].

**Figure 2:**
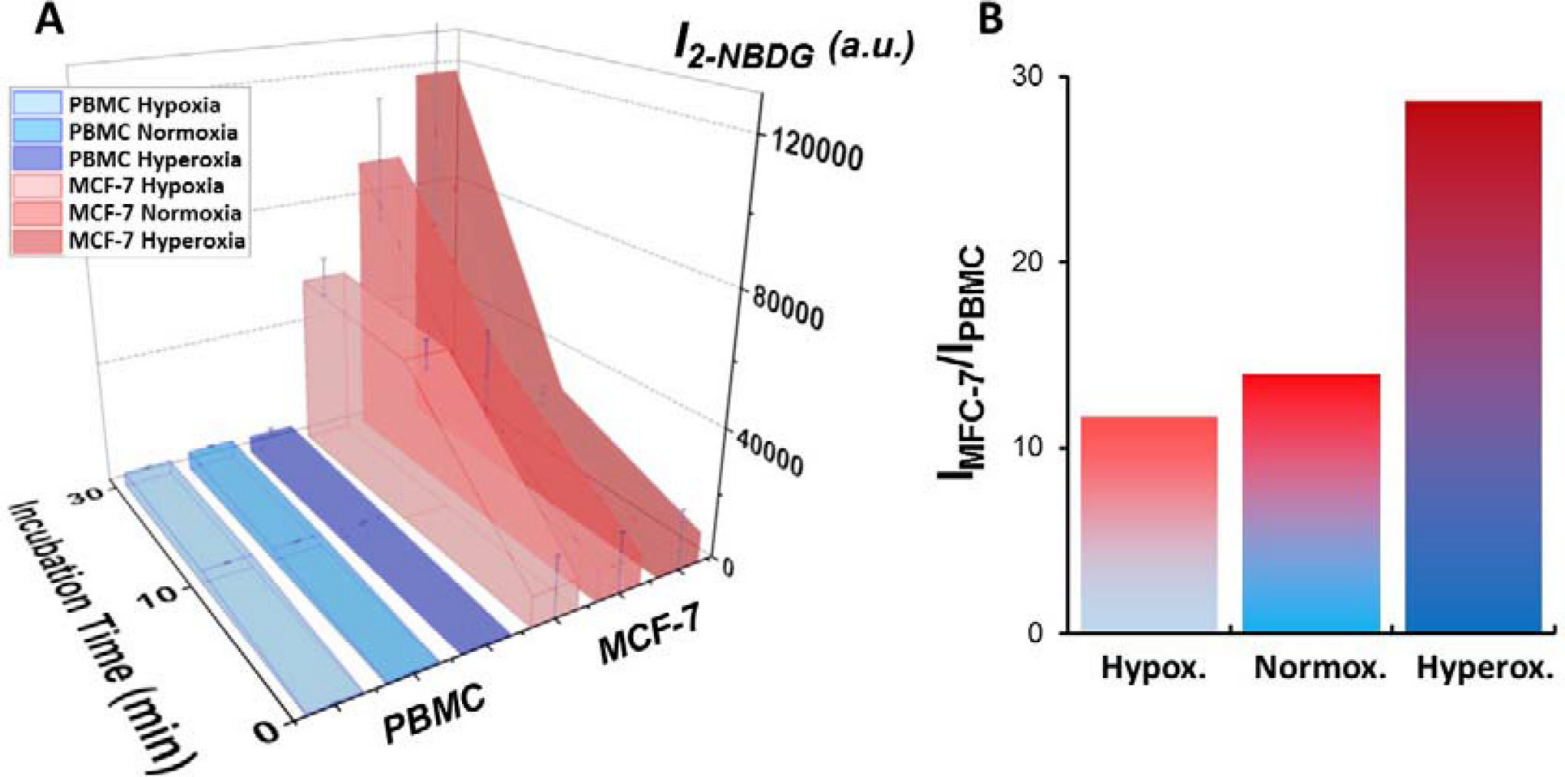
(**A**) Optimization of 2-NBDG incubation time under different microenvironments: hypoxia, normoxia, and hyperoxia. 3D Walls graph with the oxygen condition for each cell line in *z-axis*, incubation time (min) in *x-axis*, and intensity of 2-NBDG/a.u. in *y-axis*. The results are presented as mean ± standard deviation of three independent experiments (*n* = 3) for samples containing cell ratios of 1:10 MCF-7:PBMC. The median values of 10,000 cytometry events were recorded for each sample. The Kruskal-Wallis test revealed a significant difference between cancer (MCF-7) and normal (PBMC) cells in optimized conditions, *p* < 0.01. (**B**) Ratiometric difference in 2-NBDG fluorescence between PBMC and MCF-7 obtained by dividing the signal intensity of the tumor cell by that of the normal cells as a function of the oxygen conditions.

**Figure 3:**
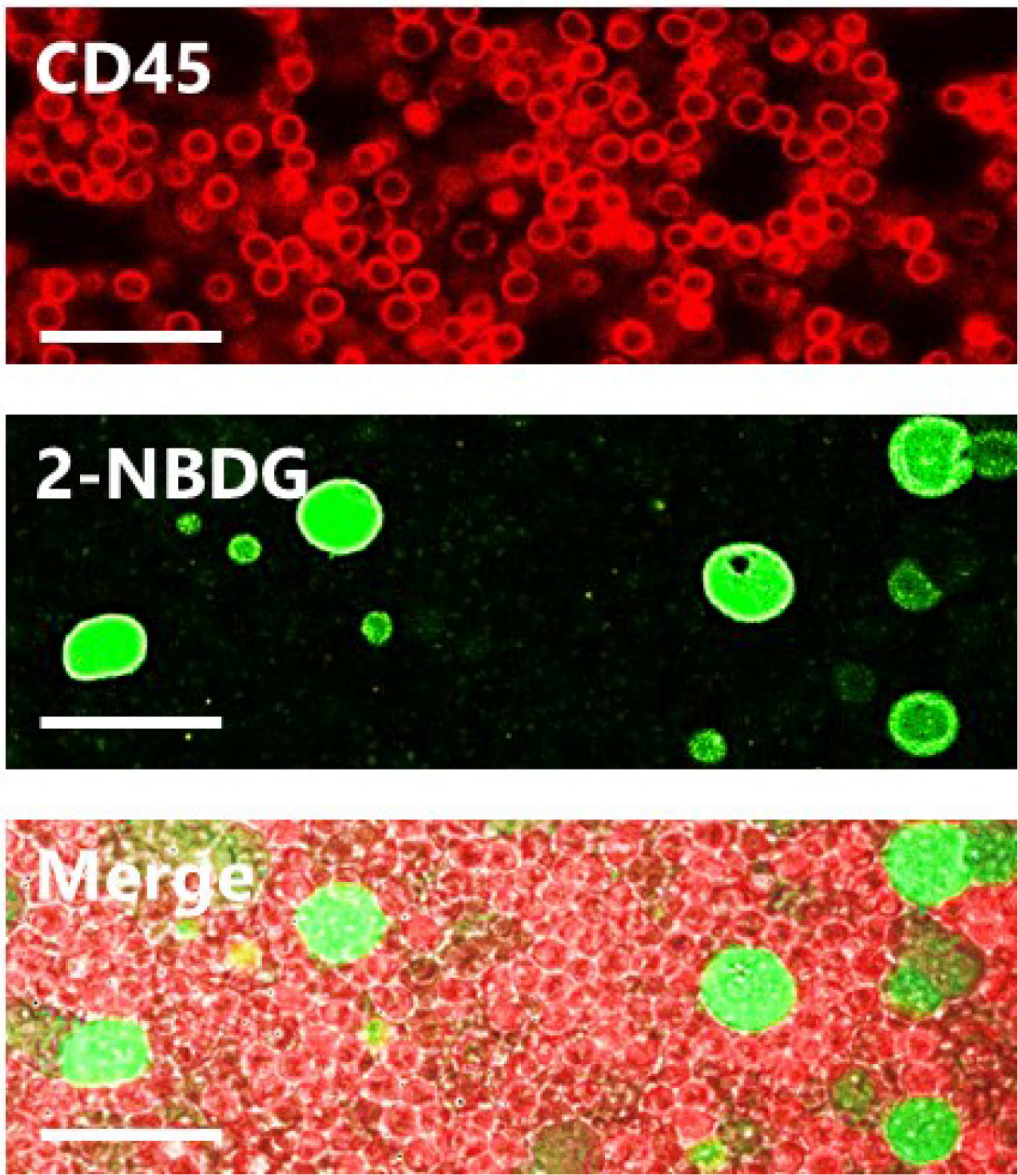
Confocal fluorescence microscopy images of 2-NBDG uptake for PBMC and MCF-7 under optimized conditions for samples containing cell ratios of 1:10 MCF-7:PBMC. Scale bars = 40 μm

Several experimental parameters (incubation time, ionic strength, pH, temperature and oxygen content) were investigated to maximize the difference in fluorescence emission between healthy and tumor cells. Notably, alterations of ionic strength, pH, and temperature did not produce any relevant effect. Regarding the incubation time, no plateau was reached within 30 minutes. However, larger incubation times did increase the fraction of cells undergoing apoptosis or autophagy due to the depletion of growth factors [21]. In fact, 2-deoxy-D-glucose (2DG), the non-fluorescent form of 2-NBDG, induces oxidative stress and apoptosis in cancer cells [22]. More interesting is, however, the case of the oxygen concentration. In fact, in our initial screening measurements, the influence of the oxygen content on the glucose uptake was clearly visible for both PBMC and MCF-7 cells. Therefore, we designed a set of experiments performed at different incubation times (from 0 to 30 min) and different oxygen concentration (hypoxia, normoxia, and hyperoxia) with the aim of understanding the simultaneous effect of these parameters on both PBMC and MCF-7 cells. Overall, in the investigated period, fluorescence displays a general increase with both time and oxygen content (Figure 2A). For all the cases, no cell death was observed within the maximum experimental period (30 min). For PBMC, fluorescence increases slightly over time. Also, the concentration of oxygen in the sample does not have any relevant effect on the emission signal. For MCF-7, although fluorescence increases considerably with time both in hypoxia and normoxia, in the case of hyperoxia such increase is significantly larger. Figure 2B shows the ratiometric difference in 2-NBDG fluorescence emission between PBMC and MCF-7 obtained by dividing the signal intensity of the tumor cells by that of the healthy ones as a function of the oxygen conditions. These results were corroborated by confocal fluorescence imaging (Figure 3). In this later panel, a remarkable increase in the intensity of the cells can be observed as compared with Figure 1B.

Incubation of cancer cells in the absence of oxygen has been frequently employed in the literature for the culture of tumor cells in order to expand them or even to establish a cell line [23, 24]. Contrarily, hyperoxia is rarely employed or studied. One reason is that long-term treatment in hyperoxia has shown to have adverse effects on cells [25, 26]. As opposed to what was hypothesized until recently, the Warburg effect is not explained by the increase of glycolysis rate of tumor cells due to mitochondrial damage. Rather, such increase is ascribed to their high replication rate, a process that requires more biomass synthesis than energy. This characteristic is common to all tumor cells that, despite their enormous heterogeneity, share the property of dedicating the resources of the tricarboxylic acid cycle to the synthesis of biomolecules [27, 28]. Thus, the larger increase of fluorescence intensity in MCF-7 cells under high oxygen content may be explained considering that hyperoxia competes with this biosynthetic flux giving rise to a compensatory increase in glycolysis. On the other hand, the replication rate of normal cells is low and, thus, the need for new biomass is limited.

**Figure 4:**
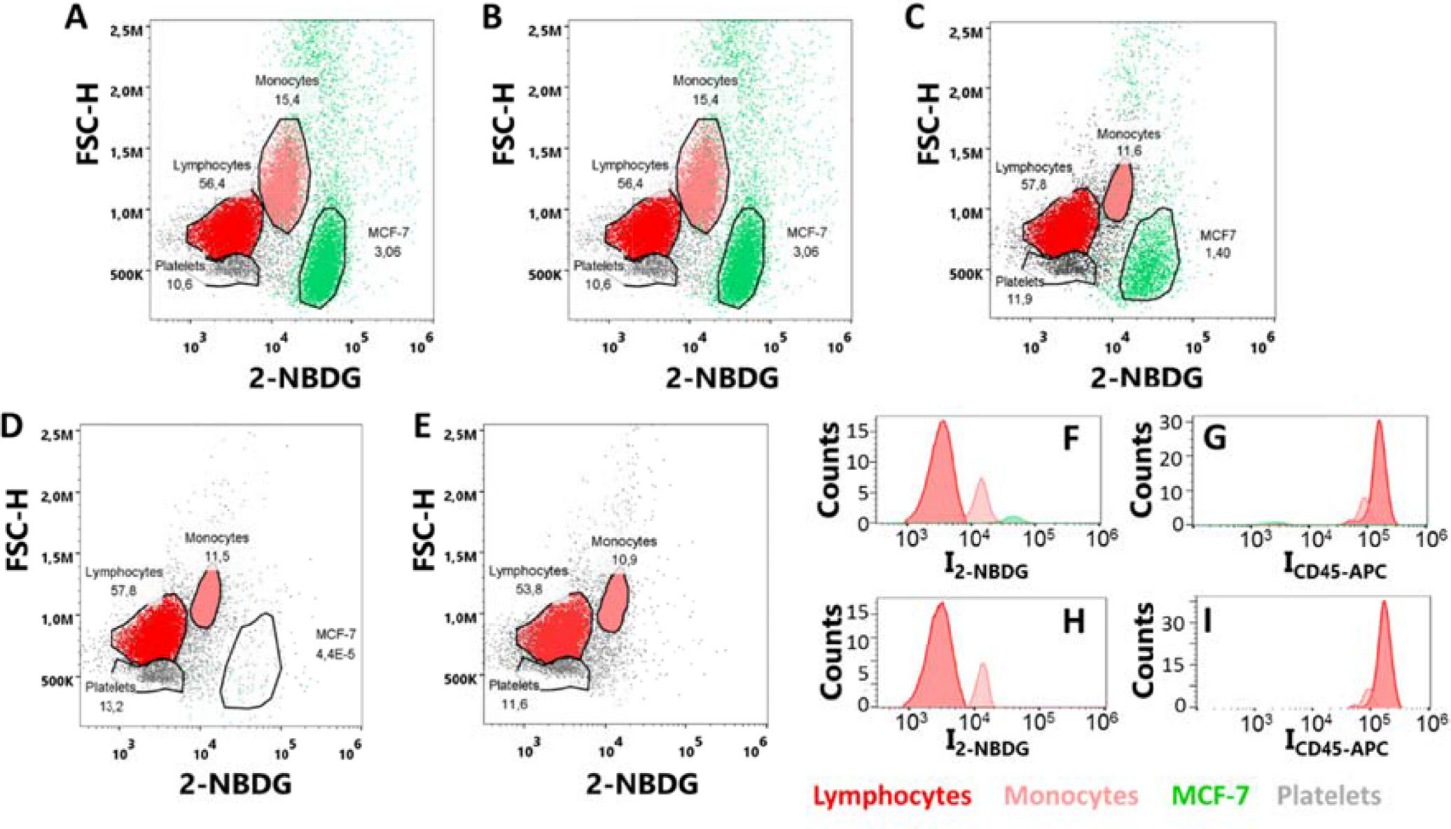
(**A**–**E**) Flow cytometry plots of MCF-7 and PBMCs samples with cell ratios (A) 1:10, (B) 1:100, (C) 1:1000, (D) 1:10000 and (E) only PBMCs; upon incubation with 2-NBDG and CD45-APC for 30 minutes under hyperoxia conditions. (**F**–**I**) Distributions of fluorescence intensities for (F) 2-NBDG and (G) CD45-APC, in a sample with a 1:1000 MCF-7:PBMC ratio; and (H) 2-NBDG and (I) CD45-APC, in a sample of PBMC. Over 10^6^ single-cell events were collected for each experiment.

**Figure 5:**
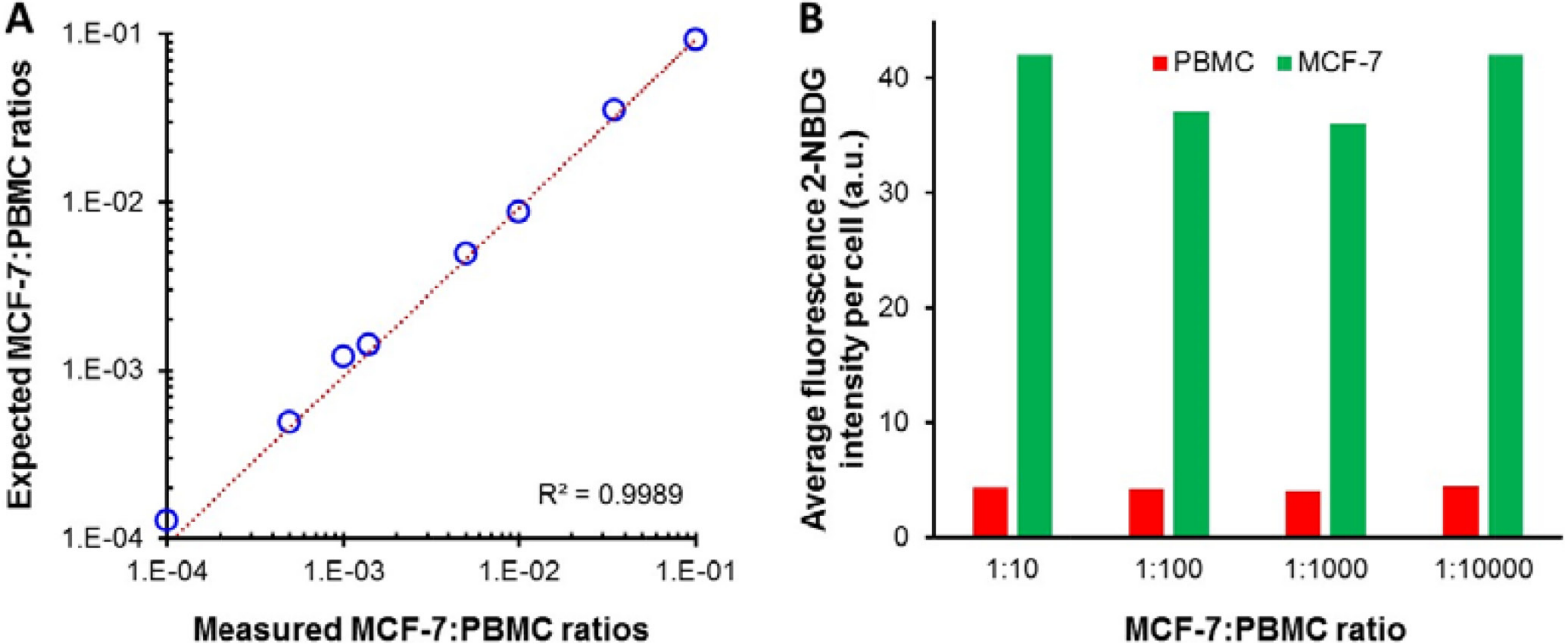
(**A**) Comparison between the expected and measured MCF-7:PBMC ratios for the samples incubated with 2-NBDG and CD-45 for 30 min under hyperoxia. (**B**) Normalized fluorescence intensities per cell for PBMC and MCF-7 treated with 2-NBDG under optimized oxygen content and incubation time conditions. Samples with cell ratios of 1:10, 1:100, 1:1000, and 1:10000.

Based on the previous results, we selected 30 min of incubation time and hyperoxia as the optimized conditions for the rest of the study. Then, in the next step, we analyzed samples prepared by diluting tumor cells into suspensions of peripheral blood mononulear cells (PBMCs). Specifically, MCF-7 cells were spiked into PBMCs cells at ratios between 1:1 and 1:10,000, and the assay was performed with the optimized conditions (i.e., 300 μM 2-NBDG in phosphate buffered saline, PBS, for 30 minutes under hyperoxia). The range of investigated cell ratios was determined down to 1:10,000 to simulate the frequency of CTCs in blood samples from patients after enrichment steps, for instance after applying a Ficoll gradient step. Suspensions of MFC-7 and PBMC cells at different ratios were then mixed with 2-NBDG and, also, CD45-APC, to additionally recognize PBMCs under hyperoxia. After 30 minutes of incubation, the samples were washed and measured in the flow-cytometer. A blue (488 nm) and a red (640 nm) lasers were used to detect either the MCF-7 (blue) or the PBMC (red) cells, as shown in Figure 4. Positive events were considered for cells showing only green fluorescence. On the other hand, cells displaying red or both green and red fluorescence were considered as negative events (PBMC). Notably, the data show a very good agreement with the expected number of cells in the samples (Figure 5A) by monitoring the emission produced during the cell metabolism of 2-NBDG upon oxygen enrichment. In this regard, it is possible to extract, for any cell ratio, an average signal for the intrinsic fluorescence developed by individual cells. This can be achieved by dividing the total fluorescence intensity, resulting from the treatment of PBMC and MCF-7 cells with 2-NBDG, by the corresponding number of events for every cell line in each sample. These results are plotted in Figure 5B. Outstandingly, these data show that at any cell ratio the fluorescence of tumor cells is considerably larger than that of normal cells, allowing for single events recognition even at highly diluted regimes.

In summary, we have demonstrated the feasibility of using 2-NBDG to discriminate tumor cells from normal cells in a flow cytometric assay under optimized experimental conditions (incubation time and hyperoxia). This method paves the way for the development of the next generation of liquid biopsies as it relies on the intrinsic and universal metabolic property of the tumor cells, rather than on a phenomenological characteristic, such as the presence of a receptor on a tumor cell membrane. We envision that this metabolic alternative to receptor-based liquid biopsies will help to solve the problems derived from the cell heterogeneity resulting from the epithelial-mesenchymal-transition. In fact, the possibility of also detecting CTCs belonging to the mesenchymal lineage, will increase the sensitivity and, thus, allow their potential detection even at earlier stages of the disease.

## MATERIALS AND METHODS

### Cell cultures

MCF-7 cancer cells, derived from mammary breast were obtained from the American Tissue Culture Collection (ATCC, Manassas, VA, USA) and cultured in Eagle's Minimum Essential Medium (EMEM) with 10% fetal bovine serum (FBS) and 0.01 mg/ml human recombinant insulin. Cells were maintained at 37°C in a humidified 5% CO2 environment.

### Blood samples and peripheral blood mononuclear cells extraction

Blood samples were obtained from healthy donors and processed within the following 24 h. 8 mL of blood from each donor were diluted 1:2 with Hank's balanced salt solution (HBSS) and disposed carefully onto a 15 mL layer of Ficoll-Plaque Plus (from Ge Healthcare LifeSciences). Samples were centrifuged for 40 min at 400 g to separate blood content. Peripheral blood mononuclear cells (PBMCs) were collected and washed with 1× PBS and finally, cultured in RPMI media supplemented with 10% fetal bovine serum at 37°C, 5% CO_2_ in a humidified atmosphere.

### 2-NBDG and CD45-APC labelling

Cells were harvested from culture dishes using trypsin 0.25% EDTA for 5 min and seeded onto 24-well plates at 1 × 10^5^ cells/ml, overnight. On the following day, cells were washed with PBS 1× and fluorescent glucose analogue 2-(N-(7-nitrobenz-2-oxa-1,3-diazol-4-yl)amino)-2-deoxyglucose (2-NBDG) and CD45-APC (both from Thermofisher) were added at a concentration of 300 μM and 2.0 μg/mL in PBS in different oxygen environments. Hyperoxia environment was established by oxygenating 2-NBDG in PBS for 30 min and hypoxia by bubbling nitrogen into 2-NBDG solution, for 30 min. The incubation time of the cells was studied from 2.5, 5, 10, 15 to 30 min. Then, 2-NBDG uptake was stopped by removing 2-NBDG and washing the cells with ice-cold PBS three times.

### Flow cytometry

Flow cytometry of the samples was carried out in a NovoCyte Flow Cytometer (from Acea Biosciences), equipped with a 488 nm and 640 nm excitation lasers and 530/30 nm and 675/30 nm detection filters. Cytometric data were analyzed with NovoExpress and FloJo VX software. The flow cytometry measurements were performed immediately after preparation of the samples. For each measurement, data of over 10^6^ single cells events were collected.

### Laser scanning confocal microscopy analysis

MCF-7 cells were harvested from culture dishes with cell dissociation buffer and 100,000 cells were spiked into 1 × 10^6^ PBMCs. The same amounts of stains were added, as previously described, and 300 μM and 30 min of time were used for the 2-NBDG incubation under hyperoxia environment, as the optimized condition; and 10 min under normoxia were used as a non-optimized condition. After, cells were washed twice by centrifugation at 250 g for 10 min and suspended in cold-PBS. Finally, 100 μL of cell solution were disposed onto 8-well μ-Ibidi plates and analyzed under laser scanning confocal microscopy using 488 nm and 633 nm excitation lasers and 515/30 nm and 650LP filters to collect fluorescence. Same exposure conditions were applied to compare 2-NBDG fluorescence intensities between samples: 40× macrolense, 69.3% laser power, 13.20 pixel dwell and middle pinhole.

### Flow cytometry of cell mixtures

Cells were harvested using trypsin 0.25% EDTA for 5 min and centrifuged for 5 min at 400 g for MCF-7 and at 150 g for PBMC cells, and suspended in ice-cold PBS at 1 × 10^6^ cells/mL. Cells were washed by centrifugation twice and MCF-7 and PBMC were mixed together in different ratios ranging from 1:10, 1:100, 1:1000 to 1:10000. Then, 2-NBDG and CD45-APC were added, then samples ewere incubated under hyperoxia for 30 min. Finally, cells were washed three times with PBS and analyzed using a NovoCyte Flow Cytometer (from Acea Biosciences) equipped with 488 nm and 640 nm excitation lasers and 530/30 nm and 675/30 nm detection filters. Flow cytometry data were analyzed and processed with FlowJo X software.

### Statistical analysis

Statistical analysis was performed to check whether intensity differences between cell line, oxygen condition, and incubation time were significant. A total of 10,000 cytometric events (*n* = 10,000) were collected per sample. To compare a quantitative variable (fluorescence intensity) *vs* qualitative variable with three factors or more (oxygen environment and cell line, three factors each) two tests may be used depending on the normality of the data: One Way ANOVA must be chosen in case of normal distribution, and the Kruskal-Wallis test in case of non-normal distribution. We tested the normality of the data with the Kolmogorov-Smirmow test and found non-normal distributions (*P* < 0.01) in all cases. Hence, the data were presented as medians and Kruskal-Walls was selected to study the significance for comparison of more than 2 non-parametric groups. Dunn's test was the *post hoc* test selected to compare all the possible pairs in each point in time. Notice that the same analysis was done for each incubation time. SPSS 22 software and a significancy of *P* < 0.01 were used.

## Abbreviations

CD45: anti-Protein tyrosine phosphatase
CTC: circulating tumor cells
HER: epidermal growth factor receptor
EGFR: epidermal growth factor receptorl
HER2: human epidermal growth factor receptor 2l
HER3: human epidermal growth factor receptor 3l
GD2: disialoganglioside GD2
PSAm: prostate-specific antigen membrane
PDGF: platelet-derived growth factor
FDA: food and drug administration
EpCAM: epithelial cell adhesion molecule
EMT: epithelial-mesenchymal transition
PET: positron emission tomography
18F-FDG: 2-[18F]fluoro-2-deoxy-D-glucose
2-NBDG: 2-[N-(7-nitrobenz-2-oxa-1,3-diazol-4-yl)amino]-2-deoxy-D-glucose
MCF-7: Michigan cancer foundation-7 cell line
MCF-12A: Michigan cancer foundation-12A cell line
BEAS-2B: human bronchial epithelial cell line
ATCC: American type culture collection
GLUT1: glucose transporter 1
PBS: phosphate buffered saline
DAPI: 4’,6-diamino-2-fenilindol
APC: allophycocyanin
EMEM: eagle's minimum essential medium
FBS: fetal bovine serum
DMEM: Dulbecco's modified eagle's medium
F12: Ham's F12 medium
BEGM^™^: bronchial epithelial growth media
EDTA: ethylenediaminetetraacetic acid
FITC: fluorescein isothiocyanate

## References

[1] Pallaoro A, Hoonejani MR, Braun GB, Meinhart CD, Moskovits M (2015). Rapid Identification by Surface-Enhanced Raman Spectroscopy of Cancer Cells at Low Concentrations Flowing in a Microfluidic Channel. ACS Nano.

[2] Alix-Panabieres C, Pantel K (2014). Technologies for detection of circulating tumor cells: facts and vision. Lab Chip.

[3] Joosse SA, Gorges TM, Pantel K (2015). Biology, detection, and clinical implications of circulating tumor cells. EMBO Mol Med.

[4] Pelaz B, Alexiou C, Alvarez-Puebla RA, Alves F, Andrews AM, Ashraf S, Balogh LP, Ballerini L, Bestetti A, Brendel C, Bosi S, Carril M, Chan WC (2017). Diverse Applications of Nanomedicine. ACS Nano.

[5] Grimm D, Bauer J, Pietsch J, Infanger M, Eucker J, Eilles C, Schoenberger J (2011). Diagnostic and therapeutic use of membrane proteins in cancer cells. Curr Med Chem.

[6] Baccelli I, Schneeweiss A, Riethdorf S, Stenzinger A, Schillert A, Vogel V, Klein C, Saini M, Bauerle T, Wallwiener M, Holland-Letz T, Hofner T, Sprick M (2013). Identification of a population of blood circulating tumor cells from breast cancer patients that initiates metastasis in a xenograft assay. Nat Biotechnol.

[7] Gorges TM, Kuske A, Röck K, Mauermann O, Müller V, Peine S, Verpoort K, Novosadova V, Kubista M, Riethdorf S, Pantel K (2016). Accession of Tumor Heterogeneity by Multiplex Transcriptome Profiling of Single Circulating Tumor Cells. Clin Chem.

[8] Yu M, Bardia A, Wittner BS, Stott SL, Smas ME, Ting DT, Isakoff SJ, Ciciliano JC, Wells MN, Shah AM, Concannon KF, Donaldson MC, Sequist LV (2013). Circulating Breast Tumor Cells Exhibit Dynamic Changes in Epithelial and Mesenchymal Composition. Science.

[9] Chaffer CL, Weinberg RA (2011). A Perspective on Cancer Cell Metastasis. Science.

[10] Lecharpentier A, Vielh P, Perez-Moreno P, Planchard D, Soria JC, Farace F (2011). Detection of circulating tumour cells with a hybrid (epithelial/mesenchymal) phenotype in patients with metastatic non-small cell lung cancer. Br J Cancer.

[11] Vander Heiden MG, Cantley LC, Thompson CB (2009). Understanding the Warburg Effect: The Metabolic Requirements of Cell Proliferation. Science.

[12] Fletcher JW, Djulbegovic B, Soares HP, Siegel BA, Lowe VJ, Lyman GH, Coleman RE, Wahl R, Paschold JC, Avrill N, Einhorn LH, Suh WW, Samson'O D (2008). Recommendations on the use of F-18-FDG PET in oncology. J Nucl Med.

[13] Pellegrino T, Parak WJ, Boudreau R, Le Gros MA, Gerion D, Alivisatos AP, Larabell CA (2003). Quantum dot-based cell motility assay. Differentiation.

[14] Parak WJ, Boudreau R, Le Gros M, Gerion D, Zanchet D, Micheel CM, Williams SC, Alivisatos AP, Larabell C (2002). Cell Motility and Metastatic Potential Studies Based on Quantum Dot Imaging of Phagokinetic Tracks. Adv Mater.

[15] Zou C, Wang Y, Shen Z (2005). 2-NBDG as a fluorescent indicator for direct glucose uptake measurement. J Biochem Biophys Methods.

[16] Holliday DL, Speirs V (2011). Choosing the right cell line for breast cancer research. Breast Cancer Res.

[17] Millon SR, Ostrander JH, Brown JQ, Raheja A, Seewaldt VL, Ramanujam N (2011). Uptake of 2-NBDG as a method to monitor therapy response in breast cancer cell lines. Breast Cancer Res Treat.

[18] Subik K, Lee JF, Baxter L, Strzepek T, Costello D, Crowley P, Xing L, Hung MC, Bonfiglio T, Hicks DG, Tang P (2010). The Expression Patterns of eR, pR, HeR2, cK5/6, eGFR, Ki-67 and AR by Immunohistochemical Analysis in Breast Cancer Cell Lines. Breast Cancer (Auckl).

[19] Natarajan A, Srienc F (1999). Dynamics of Glucose Uptake by Single Escherichia coli Cells. Metab Eng.

[20] Sauer M, Hofkens J, Enderlein J (2011). Handbook of Fluorescence Spectroscopy and Imaging.

[21] Eisenberg-Lerner A, Bialik S, Simon HU, Kimchi A (2009). Life and death partners: apoptosis, autophagy and the cross-talk between them. Cell Death Differ.

[22] Zhang D, Li J, Wang F, Hu J, Wang S, Sun Y (2014). 2-Deoxy-D-glucose targeting of glucose metabolism in cancer cells as a potential therapy. Cancer Lett.

[23] Cayrefourcq L, Mazard T, Joosse S, Solassol J, Ramos J, Assenat E, Schumacher U, Costes V, Maudelonde T, Pantel K, Alix-Panabières C (2015). Establishment and Characterization of a Cell Line from Human Circulating Colon Cancer Cells. Cancer Res.

[24] Yu M, Bardia A, Aceto N, Bersani F, Madden MW, Donaldson MC, Desai R, Zhu H, Comaills V, Zheng Z, Wittner BS, Stojanov P, Brachtel E (2014). Ex vivo culture of circulating breast tumor cells for individualized testing of drug susceptibility. Science.

[25] Allen CB, White CW (1998). Glucose modulates cell death due to normobaric hyperoxia by maintaining cellular ATP. Am J Physiol.

[26] Das KC (2013). Hyperoxia Decreases Glycolytic Capacity, Glycolytic Reserve and Oxidative Phosphorylation in MLE-12 Cells and Inhibits Complex I, II Function, but Not Complex IV in Isolated Mouse Lung Mitochondria. PLoS One.

[27] DeBerardinis RJ, Chandel NS (2016). Fundamentals of cancer metabolism. Sci Adv.

[28] Raa A, Stansberg C, Steen VM, Bjerkvig R, Reed RK, Stuhr LE (2007). Hyperoxia retards growth and induces apoptosis and loss of glands and blood vessels in DMBA-induced rat mammary tumors. BMC Cancer.

