# Correction: Metabolic pathway for the universal fluorescent recognition of tumor cells

**DOI:** 10.18632/oncotarget.27314

**Published:** 2019-11-12

**Authors:** Ana Fernandez-Carrascal, Manuel Garcia-Algar, Moritz Nazarenus, Alicia Torres-Nuñez, Luca Guerrini, Neus Feliu, Wolfgang J. Parak, Eduardo Garcia-Rico, Ramon A. Alvarez-Puebla

**Affiliations:** ^1^ Department of Physical Chemistry and EMaS, Universitat Rovira i Virgili, Tarragona, Spain; ^2^ Karolinska Institutet, Stockholm, Sweden; ^3^ Philipps-Universität Marburg, Fachbereich Physik, Marburg, Germany; ^4^ Department of Medical Oncology, Hospital Universitario HM Torrelodones, Torrelodones, Madrid, Spain; ^5^ ICREA, Barcelona, Spain

**This article has been corrected:** Due to errors in figure preparation, panels A and B of Figure 4 were accidentally duplicated. The corrected Figure 4 is shown below. The authors declare that these corrections do not change the results or conclusions of this paper.

Original article: Oncotarget. 2017; 8:76108–76115. 76108-76115. https://doi.org/10.18632/oncotarget.18551

**Figure 4:**
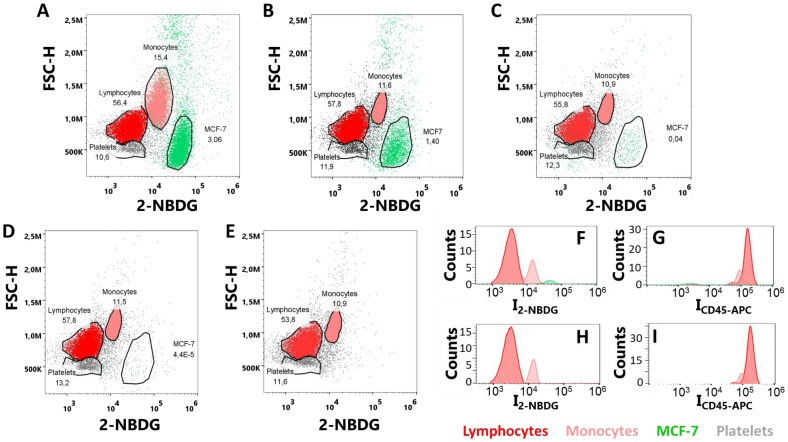
(**A**–**E**) Flow cytometry plots of MCF-7 and PBMCs samples with cell ratios (A) 1:10, (B) 1:100, (C) 1:1000, (D) 1:10000 and (E) only PBMCs; upon incubation with 2-NBDG and CD45-APC for 30 minutes under hyperoxia conditions. (**F**–**I**) Distributions of fluorescence intensities for (F) 2-NBDG and (G) CD45-APC, in a sample with a 1:1000 MCF-7:PBMC ratio; and (H) 2-NBDG and (I) CD45-APC, in a sample of PBMC. Over 10^6^ single-cell events were collected for each experiment.

